# The impact of self-efficacy on substance use in nursing students: the mediating role of sense of coherence

**DOI:** 10.1186/s12912-025-03337-w

**Published:** 2025-07-01

**Authors:** Daria Schneider-Matyka, Anna Maria Cybulska, Mariusz Panczyk, Anna Andruszkiewicz, Danuta Dyk, Aleksandra Gaworska-Krzemińska, Agnieszka Gniadek, Dorota Kozieł, Ewa Kupcewicz, Agnieszka Młynarska, Jolanta Lewko, Barbara Ślusarska, Magdalena Śniegocka, Elżbieta Grochans, Kamila Rachubińska

**Affiliations:** 1 Department of Nursing, Pomeranian Medical University in Szczecin, Szczecin, Poland; 2 Department of Education and Research of Health Sciences, Warsaw University, Warsaw, Poland; 3 Department of Basic Clinical Skills and Postgraduate Education of Nurses and Midwives, Nicolaus Copernicus University in Toruń, Bydgoszcz, Poland; 4 Department of Anaesthesiology and Intensive Care Nursing, Poznan University of Medical Sciences, Poznan, Poland; 5 Institute of Nursing and Midwifery, Faculty of Health Sciences, Institute of Maritime and Tropical Medicine, Medical University of Gdańsk, Gdańsk, Poland; 6 Department of Nursing Management and Epidemiology Nursing Institute of Nursing and Midwifery Faculty of Health Sciences, Jagiellonian University Medical College, Krakow, Poland; 7 Jan Kochanowski University of Kielce Collegium Medicum, Kielce, Poland; 8 Department of Nursing, Collegium Medicum, University of Warmia and Mazury in Olsztyn, Olsztyn, Poland; 9 Department of Gerontology and Geriatric Nursing, School of Health Sciences, Medical University of Silesia, Katowice, Poland; 10 Department of Primary Health Care, Medical University Of Bialystok, Bialystok, Poland; 11 Department of Family and Geriatric Nursing, Chair of Integrated Nursing Care, Faculty of Health Sciences, Medical University of Lublin, Lublin, Poland; 12 Józef Piłsudski University of Physical Education in Warsaw, Warsaw, Poland

**Keywords:** Nursing students, Self-efficacy, Sense of coherence, Substance use, Alcohol consumption, Psychoactive substances, Mental health

## Abstract

**Background:**

Substance use among university students, particularly nursing students, poses significant health and professional risks. Alcohol and psychoactive substance use can impact academic performance and future clinical practice. Self-efficacy and sense of coherence (SOC) are psychological constructs that influence behavior and coping mechanisms. This study investigates the mediating role of SOC in the relationship between self-efficacy and substance use among nursing students.

**Methods:**

A cross-sectional observational study was conducted among 2,689 nursing students from multiple universities in Poland. Data collection utilized standardized tools, including the Sense of Coherence-29 Scale, Generalized Self-Efficacy Scale, AUDIT (Alcohol Use Disorders Identification Test), and CRAFFT (screening tool for psychoactive substance use). Statistical analyses involved mediation modeling using Hayes’ PROCESS macro, with bootstrapping (5,000 resamples) to assess indirect effects.

**Results:**

No direct relationship was found between self-efficacy and alcohol or psychoactive substance use. However, SOC mediated the relationship between self-efficacy and psychoactive substance use, particularly through the comprehensibility component of SOC. First-year students exhibited a stronger mediating effect, suggesting that early university experiences influence the interplay between self-efficacy and substance use. The mediating effect decreased in later years, indicating adaptation to academic and social environments.

**Conclusions:**

SOC, particularly comprehensibility, plays a crucial role in linking self-efficacy to substance use behaviors in nursing students. Strengthening SOC through targeted interventions may help mitigate risky behaviors and improve overall well-being. Educational programs focusing on resilience, stress management, and coherence-building strategies could be beneficial for nursing students to support their future professional roles.

**Clinical trial number:**

Not applicable.

**Supplementary Information:**

The online version contains supplementary material available at 10.1186/s12912-025-03337-w.

## Background

Drug abuse has become a major threat to the health of young people worldwide, contributing to morbidity and mortality [1]. Experimenting with alcohol and illicit substances begins as early as adolescence [2]. According to the 2019 *European School Survey Project on Alcohol and Other Drugs* (ESPAD) report, which covered 26 countries, including Poland, an average of 33% and 2.4% of students under study have tried alcohol and cannabis, respectively, for the first time under the age of 13. The most preferred alcoholic beverages for 38% of students are spirits; beer is preferred by 31%. Cannabis is the most commonly used illicit drug in Europe (16%). 60% of study participants from Poland reported easy access to alcohol, 36% reported easy access to cannabis, and 15% reported easy access to ecstasy [3]. Recent decades have seen increasing alcohol and drug intake among students as well [4–9]. Among medical students in Poland, alarming trends in alcohol consumption have been observed. Studies have shown that 30.9% of students qualified as risky drinkers according to the AUDIT test, with male gender and smoking identified as major risk factors [10]. Moreover, an international study reported high rates of psychoactive substance use among nursing students, including marijuana, cocaine, and ecstasy [11]. These data highlight the urgent need for further research on psychological factors, such as self-efficacy and sense of coherence, which may play a key role in preventing risky health behaviors in this population. Alcohol often plays an important role in the lives of young people beginning their studies [12, 13]. There is a risk of substance abuse among students due to changes in lifestyle, reduced parental support, and the occurrence of stressful situations [14]. Research results confirm that living with family while studying is a protective factor for students [6, 15]. Higher education programmes offered by universities, particularly in the field of health sciences, should provide knowledge on the health and social consequences of the use and abuse of alcohol and other psychoactive substances, as these represent major health and social problems in university life [11]. The consequences of alcohol abuse are associated with physical health problems and poor academic performance [16, 17]. The nursing profession is not immune to the effects of psychoactive substance abuse. The rates of psychoactive substance abuse among nurses are believed to mirror those in the general population [18]. The use of alcohol and other substances by nurses potentially poses a risk of injury or death to patients, the public and the nurses themselves [19]. Nursing students are future healthcare professionals and members of therapeutic teams. For this reason, substance abuse among nursing students leads to more extensive negative consequences than those for other students. Substance abuse by nursing students can adversely affect their future ability to practice their profession and limit their recognition of substance abuse-related problems in their own patients [20–22]. It is very important to identify factors influencing the use of psychoactive substances among nursing students as future healthcare professionals.

*Coherence* (SOC), according to Antonovsky, is a generalised attitude towards the world, defined as the ability to recognise life stressors and then use coping resources effectively. A strong inner sense of coherence provides people with greater strength to cope with anxiety-inducing life experiences and helps them to move through the successive stages of life with greater strength [23–25]. The sense of coherence comprises three components, i.e., the sense of comprehensibility, the sense of manageability/steerability, and the sense of meaningfulness. The *sense of comprehensibility* is related to the cognitive aspect and allows reality to be appropriately assessed and the individual life events to be perceived as a challenge that can be dealt with. The sense of comprehensibility is the extent to which a person perceives stimuli coming from the external and internal environment as clear, ordered, coherent and structured. The *sense of steerability* refers to the extent to which the perceived resources to which an individual has access are considered to be sufficient to meet the demands placed upon them. The availability of resources refers to both the person’s individual capabilities and the resources of others on which the person can rely. Moreover, in adverse situations, this provides the confidence needed to handle them effectively. The *sense of meaningfulness* concerns the emotional and motivational dimensions. It specifies the extent to which a person feels that his or her life has emotional meaning and that the problems and demands placed upon him or her are worth the effort, commitment and sacrifice [26]. Previous research indicates that selected demographic variables may influence the level of Sense of Coherence (SOC). Age has been positively associated with higher SOC levels, likely due to greater life experience and better developed coping skills [27, 28].

Similarly, academic maturity, measured by the year of study, may contribute to higher SOC through adaptation to academic demands [28]. Gender differences have also been reported — some studies suggest that male students tend to score higher in the manageability component, whereas female students score higher in meaningfulness [28, 29]. Considering these demographic factors helps to better understand the mechanisms shaping SOC during university education and their potential impact on health-related behaviors [25].

The *sense of self-efficacy* is defined as the belief that a person has the necessary skills to perform a task in order to cope with difficult life circumstances. The sense of self-efficacy is a factor contributing to a change in behaviour [30, 31]. Having a stronger sense of individual competence is associated with greater success, better health and greater social integration. The concept of self-efficacy is used in many areas, i.e., physical and mental health, emotional problems, school success, the choice of career, and social and political life. If the individual believes that the outcome will be positive according to the sense of self-efficacy, he or she acts more actively in order to take control of their life [32]. The sense of self-efficacy does not focus on people’s personal skills but on the outcomes that people can achieve through the use of these skills [33]. The perception of self-efficacy comprises information obtained from four primary sources [31]. These include events experienced directly by an individual [34], indirect experiences learned from others [33], verbal persuasion, defined as the way people convince individuals that they are able to perform a task successfully [35], and the mental state of individuals [36]. The way an individual feels physically and mentally is an element that influences the perception of self-efficacy [37]. On the other hand, the sense of self-efficacy at work is defined as the belief in one’s own efficacy in dealing with tasks, demands and stress at work [38]. The sense of self-efficacy was indicated as a factor protecting against professional burnout among professionals [38–40] and students [41].

Self-efficacy and Sense of Coherence (SOC) are particularly important psychological constructs among young adults, such as university students. During this developmental period, young people face major academic and personal challenges, and both self-efficacy and SOC serve as protective factors against maladaptive behaviors, including substance use. Higher self-efficacy levels are consistently associated with greater resilience and reduced risk of alcohol and drug use [42, 43] Likewise, a strong SOC has been shown to predict better health outcomes and lower engagement in risky health behaviors [27, 29]. Gender differences and academic maturity (measured by the year of study) may also impact these constructs. Some studies suggest that male students score higher in the manageability component of SOC, while female students often report higher scores in meaningfulness [29]. Age and educational progress have been associated with increases in SOC over time [27]. These factors justify the exploration of moderators, such as the year of study, when examining the relationship between self-efficacy, SOC, and substance use in nursing students.

The aim of this study was to examine the mediating role of coherence in the relationship between self-efficacy and substance use, and to explore whether the year of study moderates these relationships among nursing students.

## Methods

### Settings and design

The present cross-sectional observational study was conducted at a single time point at several universities (University of Warmia and Mazury in Olsztyn; Gdańsk Medical University; Collegium Medicum at the Nicolaus Copernicus University in Bydgoszcz; Medical University of Silesia in Katowice; Dąbrowa Górnicza University of Strategic Planning; Jagiellonian University Medical College; Jan Kochanowski University in Kielce; Lublin Medical University; Białystok Medical University; Warsaw University of Physical Education; Poznań University of Medical Sciences; Pomeranian Medical University in Szczecin) educating nurses throughout Poland. A total of 2,689 1st cycle programme nursing students in Poland participated in the study, which was conducted from October to December 2023.

The respondents were informed of the aim and anonymity of the study and had the opportunity to ask questions and receive comprehensive explanations. They could also opt out of the study at any time without giving a reason. The following inclusion criteria were adopted: participants had to be at least 18 years old in order to provide legally valid informed consent to participate in the study, furthermore, since the psychological constructs under investigation, namely self-efficacy and sense of coherence, are still developing during adolescence, limiting the sample to adults ensured greater theoretical consistency; 1st cycle programme nursing study; informed consent to participate in the study; the subject’s statement: no history of mental illnesses; correct and full completion of the questionnaires. The exclusion criteria adopted included the lack of informed consent to participate in the study or the subjects’ statement: diagnosed mental illness. The time allocated to complete the questionnaire was approximately 15 min. After obtaining approval from the dean’s office, trained researchers distributed paper versions of the questionnaires among nursing students.

The size of the sample study was determined based on statistical data on the number of nursing students studying in Poland in the academic year 2023/2024. In the academic year under study, 36,909 people studied in the major of nursing. The confidence level was set at 95%, the maximum error at 5%, and the estimated fraction size at 0.5. The total number of nursing students qualified to participate in the study was 381. A total of 3,000 questionnaire sets were distributed, of which 2,689 were qualified for final analysis (return rate of 89.6%). The study is part of a larger research project carried out among nursing students in Poland.

This study was designed and reported in accordance with the STROBE (Strengthening the Reporting of Observational Studies in Epidemiology) guidelines [44].

### Data collection

The study was conducted using a questionnaire technique. To collect empirical data, an original questionnaire developed by the authors was used, which included questions on socio-demographic data (i.e., age, sex, year of studies, place of residence) and standardised research tools: Sense of Coherence-29 Scale, Generalized Self-Efficacy Scale, CRAFFT and AUDIT.

The Sense of Coherence-29 scale is a 29-item questionnaire developed by Antonovsky in 1983 to determine the coherence level. It comprises three components: SOC 1, which measures comprehensibility, SOC 2 – manageability, and SOC 3 – meaningfulness. The SOC-29 includes 29 items scored on a 7-point semantic differential scale. Total scores range from 29 to 203, with higher scores indicating a stronger sense of coherence. The internal consistency coefficients, calculated by the split-half method with Spearman-Brown correction, amounted to 0.92 for the sense of coherence, 0.78 for the sense of comprehensibility, 0.72 for the sense of manageability, and 0.68 for the sense of meaningfulness, respectively [45].

The Generalised Self-Efficacy Scale refers to the concept of expectations concerning self-efficacy, formulated by Bandura (1977, 1997). The GSES consists of 10 items rated on a 4-point Likert scale (1 = Not at all true to 4 = Exactly true). Higher total scores (ranging from 10 to 40) indicate higher perceived self-efficacy. The alpha Cronbach values ranged from 0.76 to 0.90, with the majority amounting to approximately 0.80 [46].

AUDIT has been the world’s most widely used screening tool for the presence of alcohol since 1989. It is available in more than 40 languages and enquires about the three key domains of: alcohol intake, potential dependence on alcohol, and experience of alcohol-related harm. The questions included in the AUDIT reflect the fundamental relationship between humans and alcohol, including its capacity to cause dependence (addition) and a number of harmful consequences. The AUDIT consists of 10 items rated on a combination of frequency scales and quantity scales. Total scores range from 0 to 40, with higher scores indicating more hazardous or harmful alcohol use [47].

CRAFFT is a screening tool designed to identify the use of psychoactive substances, risks associated with driving, and disturbances related to substance use among young people. It is the best-tested screening tool for the investigation of psychoactive substance use by young people. The CRAFFT is a 6-item screening tool with yes/no response options. A total score of 2 or more indicates a high risk of problematic substance use [48].

### Data analysis

For all continuous variables, the following were calculated: the mean (M), standard deviation (SD), median (ME), semi-interquartile range (IQR/2), and the minimum and maximum values (Min. – Max.). Categorical variables are presented using frequencies and percentages.

The quantitative relationship between the independent variable and the dependent variable was analysed using linear regression analysis in a mediation model to determine the mediating effect of stress on the relationship between the sense of coherence and the sense of self-efficacy, as well as the use of alcohol and psychoactive substances. Mediation analysis was carried out using a method developed by Hayes (2018) [49]. This approach was used to determine whether coherence mediates the relationship between the sense of self-efficacy and the use of alcohol and psychoactive substances. The mediation model included an independent variable (X: the sense of self-efficacy), the mediator (M: coherence), and a dependent variable (Y: the use of alcohol or psychoactive substances). In order to estimate the significance of the mediation effect, bootstrapping with 5,000 re-samples to generate 95% confidence intervals for the indirect effect was used. In addition to calculating the significance of the mediation effect, the study also calculated the percentage of the overall effect that was mediated. This was accomplished by dividing the indirect effect by the overall effect and multiplying it by 100 to express it as a percentage. This enables the quantification of the extent to which the relationship between the independent variable and the dependent variable is explained by the mediator [50].

Statistical analyses were carried out using the STATISTICA program ver. 13.3 (TIBCO, Palo Alto, CA, USA). For all statistical tests, a significance level of *p* < 0.05 was adopted, meaning that results were considered statistically significant if the probability of observed differences or relationships was less than 5%.

### Ethical considerations

The study was conducted in accordance with the Declaration of Helsinki after receiving approval from the Research Ethics Committee of the Pomeranian Medical University in Szczecin (KB.006.139.2022/Z-10953 and KB.006.139.2022/Z-10954).

## Results

The study involved 2,689 nursing students, of whom 2,460 were female (91.48%) and 229 were male (8.52%). The mean age was 21.1 years (SD = 2.1). The majority of respondents were single (64.71%), aged 21–25 years (53.11%), second-year nursing students (40.54%), and inhabitants of a city with a population of more than 100,000 (39.42%) (supplementary material: Table S1).

Table 1 shows the descriptive statistics for each standardised questionnaire used in this study. The average level of the sense of coherence, based on the stens among the study participants, was 5.60 ± 1.18, whereas the average sense of self-efficacy, based on total scores, was 25.00 ± 2.17. The average level of coherence for the individual variables was as follows: comprehensibility at 41.68 ± 6.79; steerability at 37.51 ± 4.94; and meaningfulness at 30.71 ± 3.8. On the other hand, for the AUDIT questionnaire, the average value was 5.45 ± 5.19, whereas for the CRAFFT questionnaire, the average value was 9.59 ± 2.83.

**Table 1:** Characteristics of psychological variables for *n* = 2,785

	M	SD	Mdn	IQR/2	Min - Max	CV [%]
Sense of self-efficacy according to GSES (stens)	5.60	1.18	6.0	0.50	1–10	21.04
Sense of self-esteem according to GSES (total score)	25.00	2.17	25.0	1.00	15–42	8.69
Coherence according to SOC-29 - comprehensibility	41.68	6.79	42.0	4.50	18–77	16.28
Coherence according to SOC-29 - steerability	37.51	4.94	37.0	3.00	15–70	13.16
Coherence according to SOC-29 - meaningfulness	30.71	3.80	31.0	2.50	9–56	12.38
Alcohol use according to AUDIT (total)	5.45	5.19	4.0	2.50	0–40	95.38
Psychoactive substance use according to CRAFFT (total)	9.59	2.83	10.0	1.00	0–12	29.53

The study determined the mediating role of coherence in the components of comprehensibility, steerability and meaningfulness in the relationship between the sense of self-efficacy (GSES) and alcohol use (AUDIT) among nursing students. No mediation effect was noted for any of the coherence components analysed (*p* > 0.05) (Table 2).

**Table 2:** The mediating role of coherence (comprehensibility, steerability, meaningfulness) in the relationship between the sense of self-efficacy and the use of alcohol among nursing students

			95% CI					
Type	Effect	b	Lower	Upper	β	z	*p*-value	Mediation
**Comprehensibility**								
Indirect	GSES ⇒ SOC-29_ ⇒ AUDIT	-0.01	-0.03	0.01	0.00	-1.113	0.266	10.4%
Component	GSES ⇒ SOC-29_comp	0.58	0.36	0.80	0.10	5.244	< 0.001	-
	SOC-29_comp ⇒ AUDIT	-0.02	-0.05	0.01	-0.02	-1.139	0.255	-
Direct	GSES ⇒ AUDIT	0.08	-0.08	0.25	0.02	0.989	0.323	89.6%
Total	GSES ⇒ AUDIT	0.07	-0.09	0.24	0.02	0.879	0.380	100.0%
**Steerability**								
Indirect	GSES ⇒ SOC-29_steer ⇒ AUDIT	-0.01	-0.02	0.01	0.00	-0.694	0.488	6.5%
Component	GSES ⇒ SOC-29_ steer	-0.06	-0.22	0.10	-0.01	-0.701	0.483	-
	SOC-29_ steer ⇒ AUDIT	0.10	0.06	0.14	0.09	4.880	< 0.001	-
Direct	GSES ⇒ AUDIT	0.08	-0.09	0.25	0.02	0.948	0.343	93.5%
Total	GSES ⇒ AUDIT	0.07	-0.09	0.24	0.02	0.879	0.380	100.0%
**Meaningfulness**								
Indirect	GSES ⇒ SOC-29_mean ⇒ AUDIT	-8.97e − 4	-0.02	0.02	-2.03e − 4	-0.106	0.916	1.2%
Component	GSES ⇒ SOC-29_ mean	-0.01	-0.13	0.12	0.00	-0.106	0.915	-
	SOC-29_ mean ⇒ AUDIT	0.14	0.08	0.19	0.10	5.181	< 0.001	-
Direct	GSES ⇒ AUDIT	0.08	-0.09	0.24	0.02	0.894	0.372	98.8%
Total	GSES ⇒ AUDIT	0.07	-0.09	0.24	0.02	0.879	0.380	100.0%

A mediation analysis was then carried out of the mediating role of coherence in the components of comprehensibility, steerability and meaningfulness in the relationship between the sense of self-efficacy (GSES) and the use of psychoactive substances (CRAFFT) among nursing students. The mediation effect accounted for 47.6% of the variation in the dependent variable. The study demonstrated the presence of overall mediation in the component of comprehensibility in the relationship between the sense of self-efficacy and the use of psychoactive substances among the students under study (β = 0.00, z = 2.091, *p* = 0.037). No mediation effect was noted in the components of steerability (β = 0.00, z = 0.414, *p* > 0.05) and meaningfulness (β = 0.00, z = 0.106, *p* > 0.05) (Table 3).

**Table 3:** The mediating role of coherence (comprehensibility, steerability, meaningfulness) in the relationship between the sense of self-efficacy and the use of psychoactive substances among nursing students

			95% CI					
Type	Effect	b	Lower	Upper	β	z	*p*-value	Mediation
**Comprehensibility**								
Indirect	GSES ⇒ SOC-29_comp ⇒ CRAFFT	0.01	0.00	0.02	0.00	2.091	0.037	47.6%
Component	GSES ⇒ SOC-29_comp	0.58	0.36	0.80	0.10	5.244	< 0.001	-
	SOC-29_comp ⇒ CRAFFT	0.02	0.00	0.03	0.04	2.280	0.023	-
Direct	GSES ⇒ CRAFFT	0.01	-0.08	0.10	0.00	0.253	0.801	52.4%
Total	GSES ⇒ CRAFFT	0.02	-0.07	0.11	0.01	0.484	0.628	100.0%
**Steerability**								
Indirect	GSES ⇒ SOC-29_steer ⇒ CRAFFT	0.00	0.00	0.00	0.00	0.414	0.679	1.4%
Component	GSES ⇒ SOC-29_ steer	-0.06	-0.22	0.10	-0.01	-0.701	0.483	-
	SOC-29_ steer ⇒ CRAFFT	-0.01	-0.03	0.02	-0.01	-0.514	0.607	-
Direct	GSES ⇒ CRAFFT	0.02	-0.07	0.11	0.01	0.477	0.633	98.6%
Total	GSES ⇒ CRAFFT	0.02	-0.07	0.11	0.01	0.484	0.628	100.0%
**Meaningfulness**								
Indirect	GSES ⇒ SOC-29_mean ⇒ CRAFFT	0.00	0.00	0.00	0.00	0.106	0.916	0.6%
Component	GSES ⇒ SOC-29_ mean	-0.01	-0.13	0.12	0.00	-0.106	0.915	-
	SOC-29_ mean ⇒ CRAFFT	-0.02	-0.05	0.01	-0.03	-1.354	0.176	-
Direct	GSES ⇒ CRAFFT	0.02	-0.07	0.11	0.01	0.481	0.630	99.4%
Total	GSES ⇒ CRAFFT	0.02	-0.07	0.11	0.01	0.484	0.628	100.0%

A mediation analysis was then carried out of the role of coherence in the components of comprehensibility, steerability and meaningfulness according to SOC-29 in the relationship between the sense of self-efficacy according to GSES and alcohol use according to AUDIT, with consideration of the moderator, i.e., the year of studies for nursing students.

The mediation analysis for the first-year students revealed an interaction between the sense of self-efficacy according to GSES, the sense of coherence in the component of comprehensibility according to SOC-29, and the use of alcohol according to AUDIT (Table S2).

### Mediation component

#### Direct effect of the sense of self-efficacy according to GSES on comprehensibility according to SOC-29

A direct effect of the sense of self-efficacy according to GSES on comprehensibility according to SOC-29 was demonstrated. The direct pathway from the sense of self-efficacy according to GSES to coherence according to SOC-29, with consideration of the component of comprehensibility, was quantified with a coefficient of 0.57, and a statistically significant p-value of 0.002. This shows that higher scores for self-efficacy are related to an increased sense of coherence in the component of comprehensibility. A significant effect (β = 0.10) suggests a moderate effect of the sense of self-efficacy on comprehensibility according to SOC-29 among the first-year students.

#### Indirect effect of comprehensibility according to SOC-29 on alcohol use according to AUDIT

The study also found a mediating effect of comprehensibility according to SOC-29 on alcohol use according to AUDIT. The effect of comprehensibility according to SOC-29 on alcohol use according to AUDIT was represented by a negative coefficient of -0.09, with a highly significant p-value (< 0.001). This suggests that higher comprehensibility, according to SOC-29, leads to lower AUDIT scores, which implies smaller problems related to alcohol intake. The standardised coefficient (β=-0.11) highlights the considerable effect of coherence on behaviours related to alcohol use.

### Indirect effect (mediation pathway)

The indirect effect of the sense of self-efficacy according to GSES on alcohol use according to AUDIT through comprehensibility according to SOC-29 was negative (-0.05) and statistically significant (*p* = 0.006). This result supports the hypothesis that comprehensibility, according to SOC-29, mediates the relationship between the sense of self-efficacy and alcohol use. In particular, this effect indicates that an increased sense of self-efficacy increases comprehensibility according to SOC-29, which contributes to lower alcohol intake.

### Overall and direct effect of GSES on AUDIT

Overall effect: The overall effect of the sense of self-efficacy according to GSES on alcohol use according to AUDIT was insignificant (*p* = 0.989), with the coefficient close to zero (0.00). This indicates that considering the pathway through comprehensibility according to SOC-29, the sense of self-efficacy, according to GSES, has no direct significant effect on alcohol use according to AUDIT.

The direct effect of the sense of self-efficacy according to GSES on alcohol use according to AUDIT: The direct pathway from the sense of self-efficacy according to GSES to alcohol use according to AUDIT, excluding comprehensibility according to SOC-29, also indicated an insignificant effect (β=-0.01, z = 0.339, *p* = 0.735) and a small positive coefficient (0.05). This suggests that without the mediation of comprehensibility according to SOC-29, the sense of self-efficacy, according to GSES, has a minimal direct effect on alcohol use according to AUDIT (Table S2).

Mediation analysis for the first-year students revealed no interactions between the sense of self-efficacy according to GSES, the sense of coherence in the components of steerability and meaningfulness according to SOC-29, and alcohol use according to AUDIT (Table S2).

Mediation analysis was carried out of the role of coherence in the components of comprehensibility, steerability and meaningfulness according to SOC-29 in the relationship between the sense of self-efficacy according to GSES and the use of psychoactive substances according to CRAFFT, with consideration of the moderator, i.e. the year of studies for nursing students.

Mediation analysis for the first-year students revealed an interaction between the sense of self-efficacy according to GSES, the sense of coherence in the component of comprehensibility according to SOC-29, and alcohol use according to CRAFFT (Table S3).

### Mediation analysis of the first year of study

An analysis was carried out of the indirect effect of the sense of self-efficacy according to GSES on the use of psychoactive substances according to CRAFFT, mediated by the component of comprehensibility according to SOC-29, and whether these effects are statistically significant during the first academic year.

#### Indirect mediation effects

The effect of the sense of self-efficacy according to GSES on the component of comprehensibility according to SOC-29: The coefficient for the sense of self-efficacy according to GSES, affecting the component of comprehensibility according to SOC-29, was positive (β = 0.57, *p* = 0.002), suggesting that the higher level of the sense of self-efficacy is associated with greater comprehensibility according to SOC-29 among first-year students. This strong and significant effect shows that the sense of self-efficacy considerably improves coherence.

The effect of the sense of comprehensibility according to SOC-29 on the use of psychoactive substances according to CRAFFT: Comprehensibility according to SOC-29 had a positive effect on the use of psychoactive substances according to CRAFFT (β = 0.02, *p* = 0.006), indicating that better comprehensibility according to SOC-29 is linked to a slightly higher level of risk of psychoactive substance use. This positive relationship suggests that although comprehensibility, according to SOC-29, may generally be protective in contexts in which it leads to greater involvement in peers or social situations, it may inadvertently increase exposure to risky behaviours.

Indirect effect: The general indirect effect of the sense of self-efficacy according to GSES on psychoactive substance use according to CRAFFT through the component of comprehensibility according to SOC-29 was significant (effect = 0.01, *p* = 0.040). This suggests that the sense of self-efficacy contributes to risky behaviours indirectly through its effect on comprehensibility, according to SOC-29.

#### Direct and overall effects

Direct effect of the sense of self-efficacy, according to GSES, on the use of psychoactive substances according to CRAFFT: The direct effect of the sense of self-efficacy according to GSES on the use of psychoactive substances according to CRAFFT was not significant (β=-0.04, *p* = 0.588), indicating that the sense of self-efficacy does not contribute directly to risky behaviours, irrespective of the students’ sense of coherence.

The overall effect of the sense of coherence according to SOC-29 on the use of psychoactive substances according to CRAFFT: The overall effect, which combines both direct and indirect pathways, was also insignificant (β=-0.03, *p* = 0.706). This further emphasises the central role of the mediation pathway through comprehensibility, according to SOC-29.

### Mediation analysis of the second year of study

#### Indirect mediation effects

Effect of the sense of self-efficacy according to GSES on comprehensibility according to SOC-29: In the second year of study, the effect of the sense of self-efficacy according to GSES on comprehensibility according to SOC-29 remained strong and became even more pronounced (β = 0.74, *p* < 0.001). This indicates an increase in the effect of the sense of self-efficacy on the perception of comprehensibility as the component of coherence according to SOC-29, as compared to the first year of study (β = 0.57, *p* = 0.002).

The effect of the sense of comprehensibility according to SOC-29 on the use of psychoactive substances according to CRAFFT: The effect of the sense of comprehensibility according to SOC-29 on the use of psychoactive substances according to CRAFFT in the second year of study was also positive (β = 0.02, *p* = 0.006), similar to the first year. The size and importance remained consistent, suggesting a stable relationship between comprehensibility and coherence according to SOC-29 and risky behaviours during these years.

#### Indirect effect

The general indirect effect of the sense of self-efficacy according to GSES on the use of psychoactive substances according to CRAFFT through comprehensibility according to SOC-29 was slightly more significant in the second year (effect = 0.02, *p* = 0.020), as compared to the first year (effect = 0.01, *p* = 0.040). This indicates the increasing contribution of the sense of self-efficacy through the mediation of the coherence perceived for risky behaviours.

#### Direct and overall effects

Direct effect of the sense of self-efficacy according to GSES on the use of psychoactive substances according to CRAFFT: The direct effect remained insignificant in the second year (β = 0.04, *p* = 0.572), in line with the first year (β=-0.04, *p* = 0.588), indicating that the effect of the sense of self-efficacy on the use of psychoactive substances according to CRAFFT is mediated by comprehensibility according to SOC-29 in both academic years.

The overall effect of the sense of self-efficacy according to GSES on the use of psychoactive substances according to CRAFFT: The overall effect in the second year was slightly more positive (β = 0.06, *p* = 0.424) compared to the first year (β=-0.03, *p* = 0.706), while still remaining insignificant. This slight increase may suggest an evolving contribution of the sense of self-efficacy to risky behaviours as the academic career progresses.

Increased mediating role: The second year of the study showed an increased role of comprehensibility according to SOC-29 as the mediator. This may reflect the deeper integration of coherence with the students’ coping mechanisms as they become more established in the university community.

Stability of direct effects: The direct effect of the sense of self-efficacy according to GSES on the use of psychoactive substances according to CRAFFT, which was consistently insignificant in both years, suggests that the role of the sense of self-efficacy in the direct contribution to risky behaviours related to the use of psychoactive substances is minimal.

The change from a slightly negative overall effect to a slightly positive one suggests that, as students mature and their sense of self-efficacy strengthens, their coherence in the component of comprehensibility may lead to slightly increased engagement in risky behaviours, possibly due to increased social activity or pressure.

Mediation analysis for the first-year students reveals no interactions between the sense of self-efficacy according to GSES, the sense of coherence in the components of steerability and meaningfulness according to SOC-29, and the use of psychoactive substances according to CRAFFT (Table S3).

## Discussion

A systematic review by *Lee et al.*. found that nurses are sufficiently equipped to detect and respond to problems related to the use of substances such as alcohol [51], and therefore the acquired knowledge of health behaviours also affects the risk behaviours undertaken. A qualitative study conducted among nursing students concluded that stress, social acceptance, environmental influences and the availability of alcohol are the most significant factors contributing to the intake of these substances [52]. On the other hand, another study indicated that young people beginning their studies show higher rates of alcohol intake than people of the same age who choose other non-university options [53, 54]. The results obtained in a study by *Skalska et al.*. show that the majority of the nursing students under study regularly take alcohol. In addition, many smoke tobacco as well as use cannabis [55]. In a study by *Tejedor-Cabrera et al.*., students over 25 years of age showed lower rates of exposure to alcohol and cannabis/hashish and experienced fewer consequences related to alcohol intake than students under 25 years of age, which indicates the beneficial effect of studying on health behaviours [56]. However, unhealthy habits and behaviours do not always improve during the course of study, which suggests that the knowledge and experience gained in nursing studies do not always support the development of healthy habits [57].

The present study assessed the role of coherence in the relationship between the sense of self-efficacy and the use of alcohol and psychoactive substances among nursing students. The study found no effect of the sense of self-efficacy on the use of alcohol and psychoactive substances, but it determined the mediating role of coherence in the relationship between the sense of self-efficacy and the use of alcohol and psychoactive substances among first-year students. The subsequent years of study reinforce the observations from the first year.

The year of study emerged as a significant moderator in the relationship between self-efficacy, SOC, and substance use. This finding may be explained by the gradual development of coping strategies, increased academic maturity, and exposure to health-related knowledge as students progress through the program. First-year students are typically more vulnerable to stress, uncertainty, and transitional challenges, which may contribute to lower self-regulation and a higher likelihood of engaging in risky behaviors. In contrast, senior students may have developed a more coherent sense of identity and stronger internal resources, which buffer against substance use. These findings align with previous studies emphasizing the role of academic experience in psychological adaptation [27, 29]. While the theoretical model proposed in this study assumed a clear mediating role of SOC between self-efficacy and substance use, the results partially confirmed this hypothesis. Although SOC significantly mediated the relationship, the strength of the mediation varied depending on the type of substance and the year of study. One possible explanation for these findings could be the developmental variability in coping strategies and personal resilience during early adulthood. Furthermore, cultural factors, educational stressors, and differences in health-related awareness across academic years might have influenced the constructs differently than initially anticipated. These discrepancies highlight the complexity of psychological adaptation processes among young adults and suggest that additional moderating factors should be considered in future research.

Similarly, a study by *Guldager et al.*. showed no significant effect of self-efficacy on refusal to take alcohol among students [58]. A study by *Martínez Maldonado et al.* did not demonstrate significant differences between the students’ perceived self-efficacy and alcohol intake either [59]. *Consiglio et al.* found that the nurses who scored highly in terms of the sense of self-efficacy were more inclined to overcome problems, frustrations and obstacles while being less inclined to ruminate, run out of energy and lose motivation [60].

Another study by *da-Silva-Domingues et al.* indicated a strong relationship between the sense of coherence and health behaviours, both as a factor protecting against risky behaviours, and its positive relationship with preventive and health-promoting behaviours of adolescents, young adults and university students [61]. The results of a study by *Torinomi et al.*. indicated a positive correlation between the sense of coherence and mental health [62]. A study by *Uzdil &* G*ünaydın*, conducted among nursing students, indicated a negative relationship between the sense of coherence and the sense of self-efficacy. This means that a strong sense of coherence, i.e., general life orientation, is accompanied by a low sense of self-efficacy. In contrast, a high sense of coherence was linked to an increase in mean scores in the same group of students [63]. A study by *Kösler et al.*, conducted in Germany among 27,000 students, found a positive correlation between the sense of coherence and the sense of self-efficacy and between these two constructs and mental health and well-being [64]. The sense of coherence is considered a variable that influences people’s health. It represents a protective factor against stress and a health-promoting buffer directly related to the ability to use coping strategies in order to improve stress management [65–67].

This is supported by the results of a previous study by the current authors which indicated that the ability to assess reality reinforces the effect of the sense of self-efficacy on the use of psychoactive substances. A study by *Betke et al.*., conducted among nurses, confirmed the importance of coherence as a health potential in a stressful work environment, as a high sense of coherence translated into better mental health, proper functioning in the work environment, and the use of adaptive coping strategies. Nurses with a stronger sense of coherence used more adaptive coping strategies to deal with stress than those with an average or low sense [68]. A study by *Malinauskiene et al.*. also found that a strong sense of coherence as a personal trait serves the role of a buffer protecting nurses against the development of mental health problems [69]. A stronger correlation between lifestyle and coherence was also confirmed among medical and humanities students, as compared to science students [70].

In conclusion, it appears advisable to apply more emphasis on aspects that are relevant in the context of increasing and maintaining the sense of coherence and self-efficacy in different environments, including in schools, universities, counselling and therapy, as well as in everyday life. In light of the research findings to date and the results of the present study, it appears most reasonable to disseminate knowledge about mental health in general and the ways in which it can be influenced. Recent studies confirm the crucial role of self-efficacy in preventing substance use relapse among young adults [42]. Additionally, the sense of coherence has been identified as a significant protective factor that reduces the risk of substance use in adult populations [71]. These findings align with the theoretical framework adopted in the present study and further emphasize the importance of psychological resources in mitigating risky health behaviors among nursing students.

### Limitations

Several limitations should be acknowledged. First, the cross-sectional design of the study precludes conclusions about causal relationships between self-efficacy, SOC, and substance use. Second, data were collected through self-report measures, which are susceptible to biases such as social desirability or recall bias. Third, the sample consisted exclusively of nursing students, which limits the generalizability of the findings to other student populations or young adults in general. Fourth, the study did not utilize longitudinal data, so it remains unclear how self-efficacy and SOC evolve over time. Finally, the cultural context of Poland may influence the interpretation of the results, and caution should be exercised when applying these findings to different cultural settings.

## Conclusions

It was concluded that for first-year students, the sense of self-efficacy affects the behaviours related to the use of alcohol and psychoactive substances, primarily through the effect of comprehensibility, which denotes the ability to assess reality in an appropriate manner. While the sense of self-efficacy alone has no direct effect on the use of psychoactive substances, the effect of comprehensibility, the component of coherence, plays a crucial role. The results from the second year of studies reinforce the observations obtained from the first year and emphasise the crucial role of coherence in the relationship between the sense of self-efficacy and risky behaviours. As students progress through university life, the ability to make an accurate assessment of reality affects their sense of self-efficacy in relation to risky behaviours undertaken.

## Electronic supplementary material

Below is the link to the electronic supplementary material.

Supplementary Material 1

## Data Availability

The dataset is available upon request from the corresponding author at the email address: anna.cybulska@pum.edu.pl.

## Abbreviations

SOC: Sense of Coherence
GSES: General Self-Efficacy Scale
AUDIT: Alcohol Use Disorders Identification Test
CRAFFT: Car, Relax, Alone, Forget, Friends, Trouble- Screening tool for assessing risky psychoactive substance use in adolescents
