# Supplementary material for: The impact of self-efficacy on substance use in nursing students: the mediating role of sense of coherence
Source: BMC Nurs. 2025 Jul 1;24:809. doi: 10.1186/s12912-025-03337-w (PMC12211895; doi:10.1186/s12912-025-03337-w)
Supplement: Supplementary file 1 — Supplementary Material 1 [file 12912_2025_3337_MOESM1_ESM.docx]

**Suplementary materials**

Table S1. Study group characteristics

| **Characteristic** | **n** | **%** |
| --- | --- | --- |
| **Sex** |  |  |
| female | 2,460 | 91.48 |
| male | 229 | 8.52 |
| **Residence** |  |  |
| village | 933 | 34.70 |
| city with a population of 10,000-100,000 | 696 | 25.88 |
| city with a population >100,000 | 1,060 | 39.42 |
| **Marital status** |  |  |
| unmarried | 1,740 | 64.71 |
| informal relationship | 837 | 31.13 |
| formal relationship | 112 | 4.17 |
| **Higher education institution** |  |  |
| University of Warmia and Mazury in Olsztyn | 110 | 4.09 |
| Gdańsk Medical University | 158 | 5.88 |
| Collegium Medicum at the Nicolaus Copernicus University in Bydgoszcz | 249 | 9.26 |
| Medical University of Silesia in Katowice | 584 | 21.72 |
| Dąbrowa Górnicza University of Strategic Planning | 94 | 3.50 |
| Jagiellonian University Medical College | 314 | 11.68 |
| Jan Kochanowski University in Kielce | 177 | 6.58 |
| Lublin Medical University | 275 | 10.23 |
| Białystok Medical University | 196 | 7.29 |
| Warsaw University of Physical Education | 173 | 6.43 |
| Poznań University of Medical Sciences | 142 | 5.28 |
| Pomeranian Medical University in Szczecin | 217 | 8.07 |
| **Major** |  |  |
| nursing | 2,689 | 100.00 |
| **Year of studies*** |  |  |
| 1 | 1,040 | 38.68 |
| 2 | 1,090 | 40.54 |
| 3 | 559 | 20.79 |
| **Age*** |  |  |
| ≤ 20 | 1,161 | 43.18 |
| 21 - 25 | 1,428 | 53.11 |
| 26 - 30 | 90 | 3.35 |
| >30 | 10 | 0.04 |

* Note: Percentages may not add up to exactly 100% due to rounding.

Table S2. Mediation analysis of the role of coherence according to SOC-29 in the relationship between the sense of self-efficacy (GSES) and alcohol use (AUDIT), with consideration of the moderator, i.e., the year of studies for nursing students

| **Moderator** |  | | | | **95% CI** | |  | | |
| --- | --- | --- | --- | --- | --- | --- | --- | --- | --- |
| **Academic year** | **Type** | **Effect** | **Estimate** | **SE** | **Lower** | **Upper** | **β** | **z** | **p** |
| **COMPREHENSIBILITY** |  |  |  |  |  |  |  |  |  |
| 1 | Indirect | GSES ⇒ SOC-29_comp ⇒ AUDIT | -0.05 | 0.02 | -0.08 | -0.01 | -0.01 | -2.765 | 0.006 |
|  | Component | GSES ⇒ SOC-29_comp | 0.57 | 0.18 | 0.22 | 0.93 | 0.10 | 3.140 | 0.002 |
|  |  | SOC-29_comp ⇒ AUDIT | -0.09 | 0.01 | -0.12 | -0.06 | -0.11 | -5.835 | <0.001 |
|  | Direct | GSES ⇒ AUDIT | 0.05 | 0.14 | -0.23 | 0.32 | 0.01 | 0.339 | 0.735 |
|  | Total | GSES ⇒ AUDIT | 0.00 | 0.14 | -0.28 | 0.27 | -4.39e−4 | -0.014 | 0.989 |
| 2 | Indirect | GSES ⇒ SOC-29_comp ⇒ AUDIT | 0.01 | 0.01 | -0.01 | 0.04 | 0.00 | 1.230 | 0.219 |
|  | Component | GSES ⇒ SOC-29_comp | 0.74 | 0.17 | 0.41 | 1.07 | 0.13 | 4.370 | <0.001 |
|  |  | SOC-29_comp ⇒ AUDIT | 0.02 | 0.01 | -0.01 | 0.05 | 0.02 | 1.282 | 0.200 |
|  | Direct | GSES ⇒ AUDIT | 0.23 | 0.13 | -0.03 | 0.49 | 0.05 | 1.768 | 0.077 |
|  | Total | GSES ⇒ AUDIT | 0.24 | 0.13 | -0.01 | 0.50 | 0.06 | 1.876 | 0.061 |
| 3 | Indirect | GSES ⇒ SOC-29_comp ⇒ AUDIT | 0.02 | 0.02 | -0.01 | 0.05 | 0.00 | 1.186 | 0.236 |
|  | Component | GSES ⇒ SOC-29_comp | 0.30 | 0.24 | -0.17 | 0.78 | 0.05 | 1.241 | 0.215 |
|  |  | SOC-29_comp ⇒ AUDIT | 0.06 | 0.01 | 0.03 | 0.09 | 0.08 | 4.014 | <0.001 |
|  | Direct | GSES ⇒ AUDIT | -0.22 | 0.19 | -0.59 | 0.14 | -0.05 | -1.199 | 0.231 |
|  | Total | GSES ⇒ AUDIT | -0.21 | 0.19 | -0.57 | 0.16 | -0.05 | -1.099 | 0.272 |
| **STEERABILITY** |  |  |  |  |  |  |  |  |  |
| 1 | Indirect | GSES ⇒ SOC-29_steer⇒ AUDIT | -0.01 | 0.01 | -0.02 | 0.01 | 0.00 | -1.026 | 0.305 |
|  | Component | GSES ⇒ SOC-29_steer | -0.15 | 0.13 | -0.41 | 0.12 | -0.03 | -1.099 | 0.272 |
|  |  | SOC-29_steer⇒ AUDIT | 0.06 | 0.02 | 0.02 | 0.10 | 0.06 | 2.875 | 0.004 |
|  | Direct | GSES ⇒ AUDIT | 0.01 | 0.14 | -0.27 | 0.28 | 0.00 | 0.047 | 0.962 |
|  | Total | GSES ⇒ AUDIT | 0.00 | 0.14 | -0.28 | 0.27 | -4.39e−4 | -0.014 | 0.989 |
| 2 | Indirect | GSES ⇒ SOC-29_steer⇒ AUDIT | 0.00 | 0.01 | -0.03 | 0.03 | 0.00 | 0.171 | 0.864 |
|  | Component | GSES ⇒ SOC-29_steer | 0.02 | 0.12 | -0.22 | 0.26 | 0.01 | 0.171 | 0.864 |
|  |  | SOC-29_steer⇒ AUDIT | 0.12 | 0.02 | 0.08 | 0.15 | 0.11 | 5.719 | <0.001 |
|  | Direct | GSES ⇒ AUDIT | 0.24 | 0.13 | -0.01 | 0.50 | 0.05 | 1.867 | 0.062 |
|  | Total | GSES ⇒ AUDIT | 0.24 | 0.13 | -0.01 | 0.50 | 0.06 | 1.876 | 0.061 |
| 3 | Indirect | GSES ⇒ SOC-29_steer⇒ AUDIT | -0.01 | 0.03 | -0.06 | 0.05 | 0.00 | -0.199 | 0.842 |
|  | Component | GSES ⇒ SOC-29_steer | -0.04 | 0.18 | -0.38 | 0.31 | -0.01 | -0.199 | 0.842 |
|  |  | SOC-29_steer⇒ AUDIT | 0.15 | 0.02 | 0.11 | 0.19 | 0.14 | 7.484 | <0.001 |
|  | Direct | GSES ⇒ AUDIT | -0.20 | 0.19 | -0.56 | 0.16 | -0.05 | -1.076 | 0.282 |
|  | Total | GSES ⇒ AUDIT | -0.21 | 0.19 | -0.57 | 0.16 | -0.05 | -1.099 | 0.272 |
| **MEANINGFULNESS** |  |  |  |  |  |  |  |  |  |
| 1 | Indirect | GSES ⇒ SOC-29_mean⇒ AUDIT | -0.01 | 0.01 | -0.03 | 0.01 | 0.00 | -0.973 | 0.331 |
|  | Component | GSES ⇒ SOC-29_mean | -0.10 | 0.10 | -0.31 | 0.10 | -0.03 | -1.007 | 0.314 |
|  |  | SOC-29_mean⇒ AUDIT | 0.10 | 0.03 | 0.05 | 0.15 | 0.07 | 3.778 | <0.001 |
|  | Direct | GSES ⇒ AUDIT | 0.01 | 0.14 | -0.26 | 0.28 | 0.00 | 0.059 | 0.953 |
|  | Total | GSES ⇒ AUDIT | 0.00 | 0.14 | -0.28 | 0.27 | -4.39e−4 | -0.014 | 0.989 |
| 2 | Indirect | GSES ⇒ SOC-29_mean⇒ AUDIT | -8.26e−4 | 0.01 | -0.03 | 0.03 | -1.87e−4 | -0.062 | 0.951 |
|  | Component | GSES ⇒ SOC-29_mean | -0.01 | 0.10 | -0.19 | 0.18 | 0.00 | -0.062 | 0.951 |
|  |  | SOC-29_mean⇒ AUDIT | 0.14 | 0.03 | 0.09 | 0.19 | 0.10 | 5.348 | <0.001 |
|  | Direct | GSES ⇒ AUDIT | 0.25 | 0.13 | -0.01 | 0.50 | 0.06 | 1.893 | 0.058 |
|  | Total | GSES ⇒ AUDIT | 0.24 | 0.13 | -0.01 | 0.50 | 0.06 | 1.876 | 0.061 |
| 3 | Indirect | GSES ⇒ SOC-29_mean⇒ AUDIT | 0.03 | 0.03 | -0.02 | 0.09 | 0.01 | 1.198 | 0.231 |
|  | Component | GSES ⇒ SOC-29_mean | 0.17 | 0.14 | -0.10 | 0.44 | 0.05 | 1.213 | 0.225 |
|  |  | SOC-29_mean⇒ AUDIT | 0.20 | 0.03 | 0.15 | 0.25 | 0.15 | 7.754 | <0.001 |
|  | Direct | GSES ⇒ AUDIT | -0.24 | 0.19 | -0.60 | 0.13 | -0.05 | -1.286 | 0.198 |
|  | Total | GSES ⇒ AUDIT | -0.21 | 0.19 | -0.57 | 0.16 | -0.05 | -1.099 | 0.272 |

Table S3. Mediation analysis of the role of coherence according to SOC-29 in the relationship between the sense of self-efficacy (GSES) and the use of psychoactive substances (CRAFFT), with consideration of the moderator, i.e., the year of studies for nursing students

| **Moderator** |  | | | | **95% CI** | |  | | |
| --- | --- | --- | --- | --- | --- | --- | --- | --- | --- |
| **Academic year** | **Type** | **Effect** | **Estimate** | **SE** | **Lower** | **Upper** | **β** | **z** | **p** |
| **COMPREHENSIBILITY** |  |  |  |  |  |  |  |  |  |
| 1 | Indirect | GSES ⇒ SOC-29_comp ⇒ CRAFFT | 0.01 | 0.01 | 0.00 | 0.02 | 0.01 | 2.057 | 0.040 |
|  | Component | GSES ⇒ SOC-29_comp | 0.57 | 0.18 | 0.22 | 0.93 | 0.10 | 3.140 | 0.002 |
|  |  | SOC-29_comp ⇒ CRAFFT | 0.02 | 0.01 | 0.01 | 0.04 | 0.05 | 2.723 | 0.006 |
|  | Direct | GSES ⇒ CRAFFT | -0.04 | 0.08 | -0.19 | 0.11 | -0.02 | -0.542 | 0.588 |
|  | Total | GSES ⇒ CRAFFT | -0.03 | 0.08 | -0.18 | 0.12 | -0.01 | -0.378 | 0.706 |
| 2 | Indirect | GSES ⇒ SOC-29_comp ⇒ CRAFFT | 0.02 | 0.01 | 0.00 | 0.03 | 0.01 | 2.333 | 0.020 |
|  | Component | GSES ⇒ SOC-29_comp | 0.74 | 0.17 | 0.41 | 1.07 | 0.13 | 4.369 | <0.001 |
|  |  | SOC-29_comp ⇒ CRAFFT | 0.02 | 0.01 | 0.01 | 0.04 | 0.05 | 2.758 | 0.006 |
|  | Direct | GSES ⇒ CRAFFT | 0.04 | 0.07 | -0.10 | 0.18 | 0.02 | 0.565 | 0.572 |
|  | Total | GSES ⇒ CRAFFT | 0.06 | 0.07 | -0.08 | 0.20 | 0.02 | 0.799 | 0.424 |
| 3 | Indirect | GSES ⇒ SOC-29_comp ⇒ CRAFFT | 0.00 | 0.00 | 0.00 | 0.01 | 0.00 | 0.213 | 0.831 |
|  | Component | GSES ⇒ SOC-29_comp | 0.30 | 0.24 | -0.17 | 0.78 | 0.05 | 1.241 | 0.215 |
|  |  | SOC-29_comp ⇒ CRAFFT | 0.00 | 0.01 | -0.01 | 0.02 | 0.00 | 0.217 | 0.828 |
|  | Direct | GSES ⇒ CRAFFT | 0.05 | 0.10 | -0.15 | 0.25 | 0.02 | 0.527 | 0.598 |
|  | Total | GSES ⇒ CRAFFT | 0.05 | 0.10 | -0.15 | 0.26 | 0.02 | 0.532 | 0.595 |
| **STEERABILITY** |  |  |  |  |  |  |  |  |  |
| 1 | Indirect | GSES ⇒ SOC-29_steer⇒ CRAFFT | -2.63e−4 | 0.00 | 0.00 | 0.00 | -1.09e−4 | -0.160 | 0.873 |
|  | Component | GSES ⇒ SOC-29_steer | -0.15 | 0.13 | -0.41 | 0.12 | -0.03 | -1.099 | 0.272 |
|  |  | SOC-29_steer⇒ CRAFFT | 0.00 | 0.01 | -0.02 | 0.02 | 0.00 | 0.162 | 0.872 |
|  | Direct | GSES ⇒ CRAFFT | -0.03 | 0.08 | -0.18 | 0.12 | -0.01 | -0.374 | 0.708 |
|  | Total | GSES ⇒ CRAFFT | -0.03 | 0.08 | -0.18 | 0.12 | -0.01 | -0.378 | 0.706 |
| 2 | Indirect | GSES ⇒ SOC-29_steer⇒ CRAFFT | -3.74e−4 | 0.00 | 0.00 | 0.00 | -1.55e−4 | -0.170 | 0.865 |
|  | Component | GSES ⇒ SOC-29_steer | 0.02 | 0.12 | -0.22 | 0.26 | 0.01 | 0.171 | 0.864 |
|  |  | SOC-29_steer⇒ CRAFFT | -0.02 | 0.01 | -0.04 | 0.00 | -0.03 | -1.584 | 0.113 |
|  | Direct | GSES ⇒ CRAFFT | 0.06 | 0.07 | -0.08 | 0.20 | 0.02 | 0.804 | 0.421 |
|  | Total | GSES ⇒ CRAFFT | 0.06 | 0.07 | -0.08 | 0.20 | 0.02 | 0.799 | 0.424 |
| 3 | Indirect | GSES ⇒ SOC-29_steer⇒ CRAFFT | -2.27e−5 | 0.00 | -8.26e−4 | 0.00 | -9.43e−6 | -0.056 | 0.956 |
|  | Component | GSES ⇒ SOC-29_steer | -0.04 | 0.18 | -0.38 | 0.31 | -0.01 | -0.199 | 0.842 |
|  |  | SOC-29_steer⇒ CRAFFT | 0.00 | 0.01 | -0.02 | 0.02 | 0.00 | 0.058 | 0.954 |
|  | Direct | GSES ⇒ CRAFFT | 0.05 | 0.10 | -0.15 | 0.26 | 0.02 | 0.532 | 0.595 |
|  | Total | GSES ⇒ CRAFFT | 0.05 | 0.10 | -0.15 | 0.26 | 0.02 | 0.532 | 0.595 |
| **MEANINGFULNESS** |  |  |  |  |  |  |  |  |  |
| 1 | Indirect | GSES ⇒ SOC-29_mean⇒ CRAFFT | 0.00 | 0.00 | 0.00 | 0.00 | 0.00 | 0.348 | 0.728 |
|  | Component | GSES ⇒ SOC-29_mean | -0.10 | 0.10 | -0.31 | 0.10 | -0.03 | -1.007 | 0.314 |
|  |  | SOC-29_mean⇒ CRAFFT | -0.01 | 0.01 | -0.03 | 0.02 | -0.01 | -0.371 | 0.711 |
|  | Direct | GSES ⇒ CRAFFT | -0.03 | 0.08 | -0.18 | 0.12 | -0.01 | -0.385 | 0.700 |
|  | Total | GSES ⇒ CRAFFT | -0.03 | 0.08 | -0.18 | 0.12 | -0.01 | -0.378 | 0.706 |
| 2 | Indirect | GSES ⇒ SOC-29_mean⇒ CRAFFT | 0.00 | 0.00 | 0.00 | 0.01 | 0.00 | 0.062 | 0.951 |
|  | Component | GSES ⇒ SOC-29_mean | -0.01 | 0.10 | -0.19 | 0.18 | 0.00 | -0.062 | 0.951 |
|  |  | SOC-29_mean⇒ CRAFFT | -0.03 | 0.01 | -0.05 | 0.00 | -0.04 | -1.821 | 0.069 |
|  | Direct | GSES ⇒ CRAFFT | 0.06 | 0.07 | -0.08 | 0.20 | 0.02 | 0.797 | 0.425 |
|  | Total | GSES ⇒ CRAFFT | 0.06 | 0.07 | -0.08 | 0.20 | 0.02 | 0.799 | 0.424 |
| 3 | Indirect | GSES ⇒ SOC-29_mean⇒ CRAFFT | -0.01 | 0.01 | -0.02 | 0.00 | 0.00 | -1.084 | 0.278 |
|  | Component | GSES ⇒ SOC-29_mean | 0.17 | 0.14 | -0.10 | 0.44 | 0.05 | 1.213 | 0.225 |
|  |  | SOC-29_mean⇒ CRAFFT | -0.03 | 0.01 | -0.06 | -0.01 | -0.05 | -2.417 | 0.016 |
|  | Direct | GSES ⇒ CRAFFT | 0.06 | 0.10 | -0.14 | 0.26 | 0.02 | 0.589 | 0.556 |
|  | Total | GSES ⇒ CRAFFT | 0.05 | 0.10 | -0.15 | 0.26 | 0.02 | 0.532 | 0.595 |
